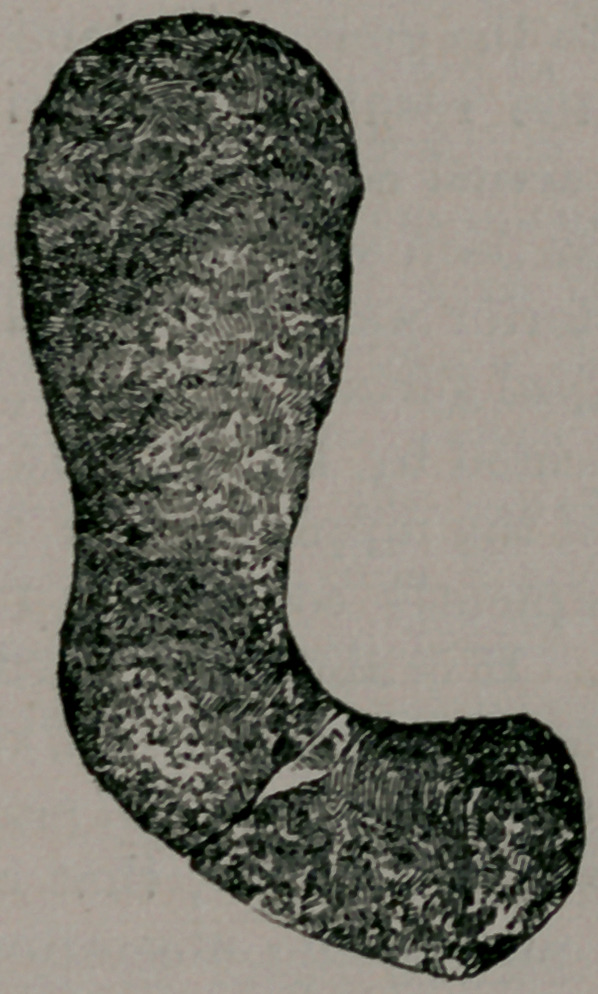# Report of a Case of Vesical Calculus—Lateral Operation—Recovery

**Published:** 1890-02

**Authors:** W. S. Elkin

**Affiliations:** Lecturer on Venereal Diseases and Genito-Urinary Surgery, Southern Medical College, Atlanta, Ga.


### Southern Medical Record.

A MONTHLY JOURNAL OF MEDICINE AND SURGERY.

VOL. XX. ATLANTA, GA., FEBRUARY, 1890. No. 2.

### Original Articles.

### REPORT OF A CASE OF VESICAL CALCULUS—LATERAL OPERATION—RECOVERY.

BY W. S. ELKIN, M. D.,

Lecturer on Venereal Diseases and Genito-Urinary Surgery, Southern Medical College, Atlanta, Ga.

On December 11, 1889, J. D., age eleven years, from Shelby county, Alabama, consulted me in reference to a severe case of cystitis that had been troubling him for the last six years. I found him weak and anæmic, presenting much physical evidence of long suffering. His mother stated that about six years ago he began to complain of a burning pain referred to the end of the penis, a frequent desire to void his urine, and other symptoms due to an inflammation of the mucous membrane of the bladder. He had been told by one physician that he had a stricture of the urethra, and by another that he had a stone in the bladder. Upon questioning the little fellow, I found that his trouble had grown much worse in the past two years, his pain being greatly increased and his strength rapidly failing. Besides the smarting pain referred to at the end of the penis, and almost constant desire to void urine, there was present in the urine pus, frequently blood, and at times pains in the scrotum, in the penis, at the base of the bladder, and along the course of the nerves of the lower extremities. H

voided urine at least every half hour during the day, and was disturbed almost every hour during the night. Walking up or down flights of steps, horseback riding, or in vehicles, greatly increased the pain. At times he found himself unable to pass his urine unless he would lie on his back or side, due to the stone dropping into the orifice of the urethra, suddenly shutting off the flow during micturition.

On December 11th, 1889, in presence of the class of the Southern Medical College and with the assistance of Drs. Gaston and Nicolson, he was anesthetized, six ounces of boracic acid solution injected into the bladder and an exploration made with Thompson's searcher. The presence of a stone was at once detected, although we were unable to determine its exact size, either by its contact with the instrument or with the finger in the rectum pressing upward on the base of the bladder and with firm pressure above and behind the symphisis pubis. We felt satisfied, however, that it was not too large to be removed by the lateral operation. As the patient had just arrived in the city, and was considerably fatigued by his long journey, the performance of the operation was postponed for a few days to give the patient rest and better prepare him for the operation.

December 14th the patient was again brought before the class. A dose of oil had been administered the night previous and two hours before the operation, and an enema of tepid water used to empty the rectum. The patient was anesthetized, placed in the lithotomy position, six ounces of boracic acid fluid injected into the bladder, and the stone again recognized with the searcher. With the assistance of Drs. Gaston, Nicolson, Olmsted and Murray I made the lateral operation, under strict antiseptics, and removed a stone weighing 460 grains. On entering the bladder with the stone forceps I came in contact with the calculus, which was presenting at the neck of the bladder just behind the prostate gland. It was easily grasped, but in endeavoring to extract it, although very gentle force was used, a part of it crushed and a portion about one-half inch in diameter, and an inch long, broke off from the nucleus and was removed.

The forceps were again introduced and an attempt made to seize the remaining portion, which was wedged above and behind pubic bone and could not be reached until I had removed the instrument and introduced my finger and dislodged it from this position. It was then readily seized in its short diameter, and extracted. This fragment, which proved to be the nucleus, was about two inches long and an inch in diameter at its greatest thickness.

The accompaning cut gives the peculiar shape of the calculus when the two fragments were put in their natural position. As the patient expressed it, when he first saw the stone, "it is shaped like a boot." The short or horizontal portion presented at the orifice of the urethra and the long or vertical portion was behind and above the pubic bone. The inorganic element composing the calculus is phosphate of lime, which accounts for its interesting and peculiar shape, since it often happens that this special form of calculi conform themselves to the shape of the bladder.

For the first twenty-four hours after the operation, there was incontinence of urine. The temperature on the second and third days was 102 1-2. After this it became normal, the wound healed rapidly by granulation, and in less than three weeks after the operation, the patient was entirely well and had returned to his home in Alabama.

## Figures and Tables

**Figure f1:**